# Environmental sustainability in veterinary clinics: best practices for the United States and Canada

**DOI:** 10.3389/fvets.2025.1690485

**Published:** 2025-12-12

**Authors:** Caroline Kern-Allely, Danielle Scott, Katie Clow, Colleen Duncan

**Affiliations:** 1 College of Veterinary Medicine and Biomedical Sciences, Colorado State University, Fort Collins,CO, United States; 2 Ontario Veterinary College, University of Guelph, Guelph, ON, Canada

**Keywords:** sustainability, veterinary medicine, clinical practice, sustainable care, environmental impact

## Abstract

**Introduction:**

Veterinary professionals in the United States and Canada are increasingly seeking ways to reduce the environmental impacts of clinical practice, reflecting a broader commitment within the profession to sustainability. While environmental sustainability frameworks are well established in human healthcare, equivalent resources for veterinary clinical practice in North America remain limited. This study aimed to develop evidence-based best practices for enhancing environmental sustainability in veterinary clinics in the United States and Canada.

**Methods:**

We conducted a gray literature review of open-access resources in veterinary medicine and human healthcare to identify explicit sustainability actions. Extracted actions were synthesized and reviewed by a panel of seven subject matter experts through a two-round modified Delphi process. Experts evaluated each action for implementation effort and environmental impact and provided qualitative feedback.

**Results:**

The final set comprised 199 actions, organized into 14 thematic categories. Experts emphasized the importance of leadership engagement, team empowerment, and balancing high-impact, resource-intensive interventions with low-effort “quick wins” to build momentum. Priority areas included energy efficiency, waste reduction (particularly anesthetic gas management), sustainable procurement, and community engagement.

**Discussion:**

The resulting framework provides a flexible, regionally relevant roadmap that clinics can adapt to their context, offering practical entry points for immediate action alongside strategies for long-term change. This resource can support veterinary teams, educators, and industry stakeholders in embedding sustainability into clinical practice, contributing to improved planetary and animal health.

## 1 Introduction

The triple planetary crises - climate change, pollution, and biodiversity loss - pose an urgent, global threat and demand sweeping, cross-sector action (1). For businesses, this means critically reviewing and refining operations to minimize environmental impacts. The healthcare industry has been particularly proactive, driven by both its substantial environmental footprint and the clear links between environmental degradation and adverse health outcomes (2). The veterinary sector shares these motivations: there is strong professional will to act (3–6), a desire for greater knowledge of sustainable veterinary practices (7), and growing evidence that veterinary clients are also seeking more sustainable options (8).

The development and dissemination of evidence-based best practices can facilitate the transition to more environmentally friendly industries. Such resources offer businesses a clear roadmap for action without the need for extensive independent research. They help prioritize steps, reduce the need for specialized expertise, and can even lower operating costs. When tailored to regional contexts, these recommendations are especially valuable, as they can account for local incentives (e.g., taxes, rebates) and the availability of specific technologies or services. In human healthcare, such evidence-based standards have been instrumental in driving meaningful, system-wide changes. Multiple organizations support sustainability efforts and provide actionable, healthcare-tailored checklists that help facilities reduce their carbon footprint, realize cost savings, and gain recognition for their achievements (9, 10).

Compared with human health, resources to guide sustainability in veterinary practice remain limited (11). While some progress has been made (e.g., Greener Practice Checklist from Vet Sustain in the UK), available action lists may overlook, or overemphasize, measures that are more relevant to specific regions. This project aimed to build upon publicly available sustainability standards in the healthcare and veterinary sectors by adapting them to the context of veterinary practices in the United States and Canada. Our objective was to establish evidence-based best practices for enhancing environmental sustainability in veterinary hospitals and clinics.

## 2 Materials and methods

### 2.1 Literature review

We conducted a review of the gray literature to identify existing best practices for sustainable business practices. We specifically searched gray literature as targeted resources were meant to be accessible to veterinary clinics and therefore must be open access and not behind a paywall. We performed two separate searches for: (a) veterinary clinical practice and (b) human healthcare. The inclusion of human healthcare ensured comprehensive coverage of sustainability practices given the *a priori* understanding that limited resources exist for veterinary medicine.

We used the following search terms for veterinary clinical practice: (veterinar* OR “vet med” OR veterinary medicine) AND (best practice OR standard OR certif* OR path* OR accredit* OR tool* OR program OR metric OR measure* OR performance OR impact OR checklist) AND (environment* (AND footprint) OR sustainab* (AND environment*) OR green OR eco OR “climate change”). Additionally, we performed a directed search of American Veterinary Medical Association (AVMA) and Canadian Veterinary Medical Association (CVMA) websites for resources on environmental sustainability.

Our search terms for human healthcare were: (healthcare OR medicine OR medical) AND (best practice OR standard OR certif* OR accredit* OR tool* OR metric OR measure* OR performance OR impact OR checklist) AND (environment* (AND footprint) OR sustainab* (AND environment*) OR green OR eco OR “climate change”).

We conducted searches using Google.com and continued until reaching three consecutive pages of 10 hyperlinks without identifying relevant documents. Given our *a priori* knowledge of limited availability of best practices for veterinary clinical practice in the US and Canada, we included global representation in our search. For human healthcare, our inclusion criteria focused on best practices relevant to the US and Canada. Our inclusion criteria specifically sought documents that contained explicit actions detailing best practices for environmental sustainability.

From the resources included, we extracted and compiled a list of individual actions. When actions were duplicative, meaning that they were conserved across multiple informing resources, we recorded the frequency that the action was referenced and, as necessary, developed statements with language that synthesized that action. All actions were thematically grouped via co-author consensus into one of 3 tiers (Core, Clinic, Community) and further categorized into one of 14 categories based on the “Pathway to a More Sustainable Clinic” resource that was co-created by the American Veterinary Medical Association, Canadian Veterinary Medical Association, and Veterinary Sustainability Alliance. Actions that we deemed not applicable to a veterinary clinic setting were excluded.

### 2.2 Expert opinion

We convened a panel of subject matter experts (SMEs) to review the action list and participate in a relative scoring system described below. We defined SMEs as individuals who have demonstrated leadership in veterinary clinical sustainability, as evidenced by serving on focus groups and committees, publishing on this topic, public speaking, and/or job descriptions.

Given our regional focus, we determined that SMEs were not to exceed 25% outside of the US and Canada. We developed an initial list of experts based on scientific communications or professional roles in clinical veterinary medical sustainability. Each expert was contacted by email to request their participation. We also employed the peer-referential technique of snowball sampling (12, 13) and asked those contacted to suggest the names of additional experts to include in our panel of SMEs. This study was classified as exempt from full review by the Colorado State University Institutional Review Board.

We utilized a modified Delphi approach to engage experts to judge the appropriateness of actions included, identify actions missing, and objectively measure the associated effort and impact of each action. When interpreted together, the effort and impact matrix can identify actions considered easy wins or those with high potential, as well as deprioritize actions requiring increased effort with a low return of impact (14). We defined effort as the work it would be to accomplish this action, with a low effort score meaning an action that is easy to do while a high effort score means more work or cost to achieve, i.e., is more resource intensive. We defined impact as the long-term effects to help future generations meet their own needs, where a high impact score would mean the action has a large effect while a low impact score would mean the action will minimally contribute to future needs. In the first round, we sent the SMEs an Excel table with the individual actions, grouped based on the framework outlined above, with a column for the effort and another for the impact they would attribute to each action. The panel was asked to review the list and add any additional actions missing from it. SMEs submitted scores between 1 and 100 for the effort and impact of each action, with 1 indicating the lowest effort or impact and 100 indicating the highest. In addition, SMEs had open text space to make comments on all actions, as well as comments about each category and broadly about environmental sustainability in veterinary practice.

We aggregated and summarized the results from the first round of SME scoring, including any new suggested actions. SMEs provided comments for specific actions leading to appropriate revisions. In addition to categorizing actions according to the “Pathway to a More Sustainable Clinic,” we also grouped related actions into themes in this version. For example, in the Energy category, themes include “energy efficient equipment” and “energy efficient lighting,” among others. These themes were determined through discussion and consensus among co-authors.

For the second round, the revised Excel table was re-distributed to the SMEs for a final review. We asked SMEs to re-score each action for an effort and impact rating, but instead of 1–100, we asked them to assign a rating of minimal, medium, high, or maximal. We assigned a 1 to 4-point scale to reflect minimal to maximal, respectively, and performed descriptive statistics reporting average effort and impact ratings. SMEs had the opportunity to provide feedback on each action, theme, category, and generally in an open text space. All SME comments and feedback from Round 1 and Round 2 were synthesized into insights for high-level discussion points for each category.

## 3 Results

Our gray literature review identified eight veterinary medicine specific and four human healthcare specific resources (Table 1). From these resources, actions were thematically grouped into the following pathways: Core (*n* = 37), Clinic (*n* = 207), and Community (*n* = 56). Within Core, actions were further classified into the following categories: Convene (*n* = 14), Assess (*n* = 12), Reflect (*n* = 6), Plan (*n* = 5). Clinic actions were classified into the following categories: Energy (*n* = 44), Waste (*n* = 84), Water (*n* = 22), Procurement (*n* = 33), Transportation (*n* = 24). Community actions were classified into the following categories: Health (*n* = 8), Stories (*n* = 9), Connections (*n* = 9), Policies (*n* = 2), Nature (*n* = 28).

**Table 1:** List of resources identified from the gray literature review, informing the list of actions for best practices for environmental sustainability.

Source	Website
Veterinary sources	
Vet Sustain *Greener Veterinary Practice Checklist*	https://vetsustain.org/resources/vet-practice-checklist
Royal College of Veterinary Surgeons *Practice Standards Scheme Environmental Sustainability Awards*	https://www.rcvs.org.uk/setting-standards/practice-standards-scheme/pss-awards/#Sustainability
Saha et al. (23) *Sustainable practices in the animal health industry: A stakeholder-based view*	https://onlinelibrary.wiley.com/doi/full/10.1002/bse.3633
Jones and West (16) *Environmental sustainability in veterinary anaesthesia*	https://www.vaajournal.org/article/S1467-2987%2819%2930018-2/fulltext
The Veterinary Nurse *A guide to sustainability in veterinary practice*	https://www.theveterinarynurse.com/content/professional/a-guide-to-sustainability-in-veterinary-practice/
The Webinar Vet *Creating an Eco-Friendly Veterinary Clinic: Steps to a Sustainable Practice*	https://thewebinarvet.com/blog/creating-an-eco-friendly-veterinary-clinic-steps-to-a-sustainable-practice
Today’s Veterinary Practice *A Practical Approach to Sustainability in the Veterinary Clinic*	https://todaysveterinarypractice.com/practice-management/a-practical-approach-to-sustainability-in-the-veterinary-clinic/
Pets app *26 easy sustainability hacks for your veterinary clinic*	https://petsapp.com/blog/26-easy-sustainability-hacks-for-your-veterinary-clinic
Human healthcare sources	
Practice Greenhealth *Sustainability solutions for health care*	https://practicegreenhealth.org/
CASCADES *Sustainable Primary Care Toolkit*	https://cascadescanada.ca/resources/sustainable-primary-care-toolkit/
The Canadian Coalition for Green Health Care *Green Office Toolkit: For Clinicians and Office Managers*	https://greenhealthcare.ca/green-office-toolkit/
Agency for Healthcare Research and Quality *Reducing Healthcare Carbon Emissions*	https://www.ahrq.gov/sites/default/files/wysiwyg/healthsystemsresearch/decarbonization/decarbonization.pdf

Seven subject matter experts were recruited and participated. SMEs represented United States (*n* = 4), Canada (*n* = 1), United Kingdom (*n* = 1), and Australia (*n* = 1). All SMEs met the expert inclusion criteria, having demonstrated leadership in environmental sustainability as detailed above. All seven SMEs completed both rounds of review for 100% completion.

**Figure 1:**
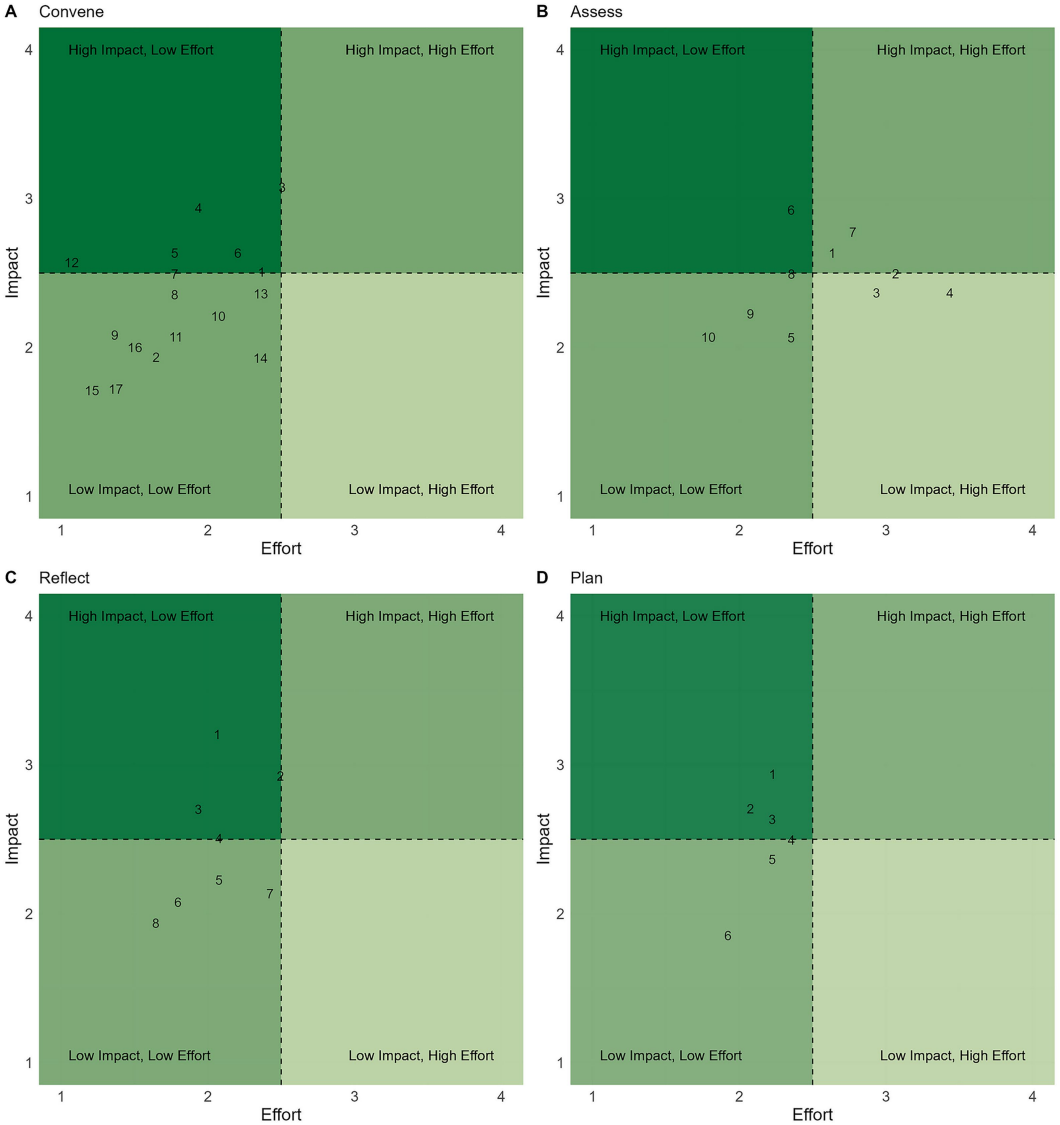
Sustainability actions under the *Core* tier are plotted based on their average effort (x-axis) and impact (y-axis) scores for the categories *Convene* **(A)**, *Assess* **(B)**, *Reflect* **(C)**, and *Plan* **(D)**. Each number corresponds to the actions listed in Tables 2–5, by referenced category.

**Figure 2:**
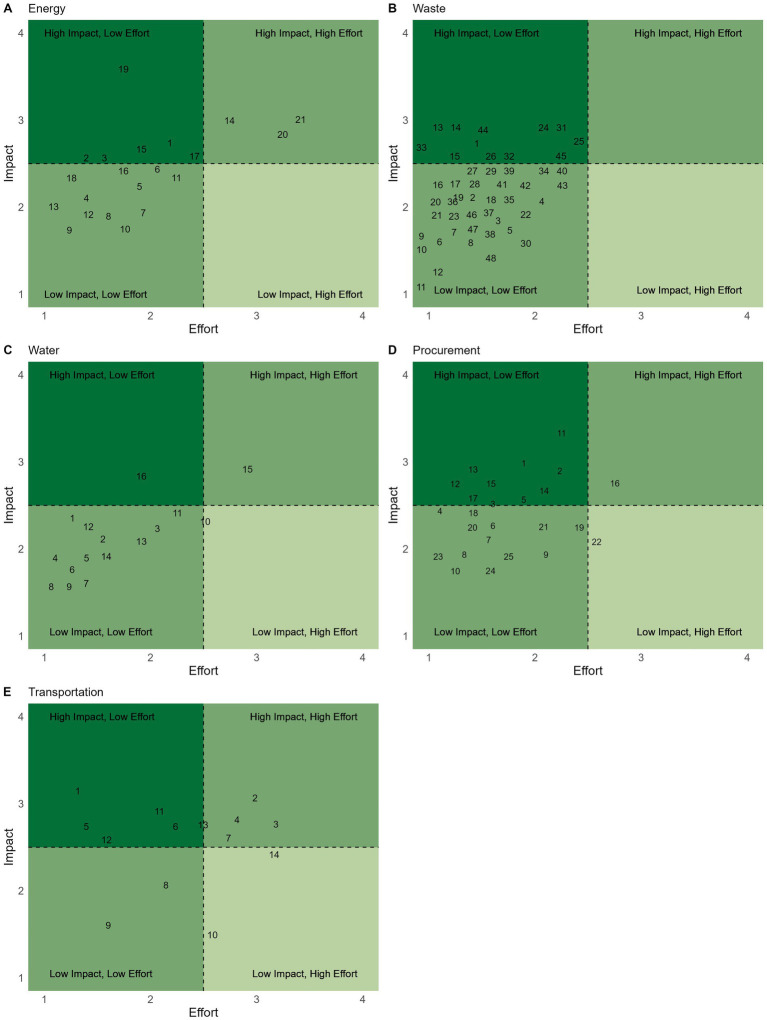
Sustainability actions under the *Clinic* tier are plotted based on their average effort (x-axis) and impact (y-axis) scores for the categories *Energy* **(A)**, *Waste* **(B)**, *Water* **(C)**, *Procurement* **(D)**, and *Transportation* **(E)**. Each number corresponds to the actions listed in Tables 6–10, by referenced category.

**Figure 3:**
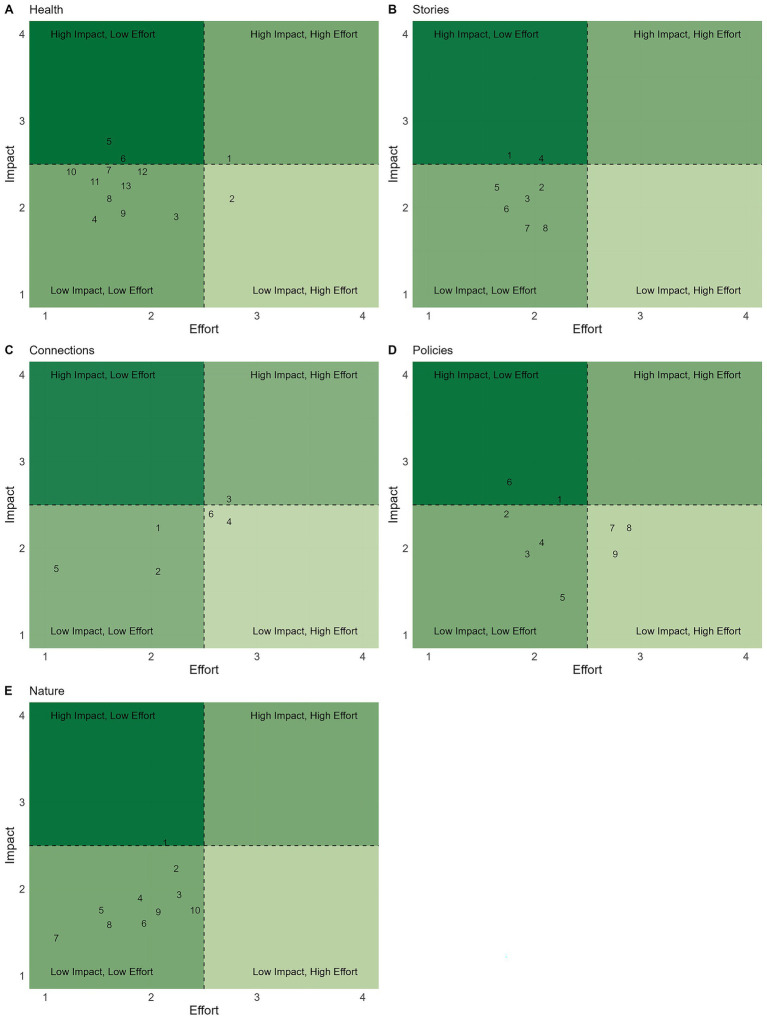
Sustainability actions under the *Community* tier are plotted based on their average effort (x-axis) and impact (y-axis) scores for the categories *Health* **(A)**, *Stories* **(B)**, *Connections* **(C)**, *Policies* **(D)**, and *Nature* **(E)**. Each number corresponds to the actions listed in Tables 11–15, by referenced category.

Based on SME feedback from Round 1, actions were revised, including indicated deletions and additions, and categorized into themes. Additionally, 36 actions were removed based on SME Round 1 recommendations. For the final review in Round 2, the total number of actions by pathway were: Core (*n* = 29), Clinic (*n* = 124), and Community (*n* = 46). No actions were added, updated or removed based on SME feedback in Round 2. The final themes and actions are presented in Tables 2–8, with associated average effort and impact scores in Figures 1–3. Further descriptive statistics are presented in Supplementary material 1.

**Table 2:** The list of actions in the *Convene* category of the *Core* tier, grouped by theme.

Theme	Number	Action
Clinic sustainability policy	1.	Create an over-arching sustainability policy detailing sustainability initiatives
	2.	Review and update sustainability policy at regular intervals
Sustainability team	3.	Engage leadership on sustainability initiatives (e.g., financial officers, biosafety/security, purchasing leads, business managers, practice owners)
	4.	Provide resources to enable sustainability team to implement initiatives
	5.	Appoint a sustainability lead or leadership team to design, implement, and manage initiatives
	6.	Measure and document sustainability team successes
	7.	Provide opportunities for all staff to contribute to sustainability group
	8.	Allocate time and space for team meetings
	9.	Outline sustainability team expectations
	10.	Hold regular sustainability meetings
	11.	Determine roles and responsibilities of sustainability team
Staff engagement in sustainability efforts	12.	Have sustainability as an agenda item at team meetings
	13.	Host games or contests for sustainability efforts
	14.	Provide compensation for staff involved in sustainability efforts
	15.	Make a sustainability ideas suggestion box
Client engagement in sustainability	16.	Include a sustainability question on client satisfaction surveys
	17.	Provide opportunities for client feedback on sustainability initiatives

Within each theme, actions are ranked by the highest impact and lowest effort. Action numbers correspond to numbers presented in Figure 1A for cross-referencing.

**Table 3:** The list of actions in the *Assess* category of the *Core* tier, grouped by theme.

Theme	Number	Action
Quantitate clinic footprint	1.	Perform an energy audit of annual energy consumption
	2.	Perform a waste audit, including universal waste and recycling
	3.	Implement ongoing process for tracing waste data volume and cost for all waste streams
	4.	Calculate total operational greenhouse gas emissions as a measure of clinic’s carbon footprint to inform and track progress
	5.	Conduct annual clinical staff commuting survey
Community sustainability resources	6.	Identify sustainability resources in your community for energy (e.g., low carbon energy providers, local renewable energy installation contractors)
	7.	Identify sustainability resources in your community for procurement
	8.	Identify sustainability resources in your community for waste (e.g., local recycling programs)
	9.	Identify sustainability resources in your community for transportation (e.g., safe bike routes)
	10.	Identify sustainability resources in your community for water (e.g., community programs geared at water reduction)

Within each theme, actions are ranked by the highest impact and lowest effort. Action numbers correspond to numbers presented in Figure 1B for cross-referencing.

**Table 4:** The list of actions in the *Reflect* category of the *Core* tier, grouped by theme.

Theme	Number	Action
Clinic tailored sustainability priorities	1.	Review sustainability data and identify priority projects for energy
	2.	Review sustainability data and identify priority projects for procurement
	3.	Create clinic SMART goals for priority projects
	4.	Review sustainability data and identify priority projects for waste
	5.	Review sustainability data and identify priority projects for transportation
	6.	Review contracts with waste contractors annually
	7.	Review broader community initiatives and identify areas for extension projects
	8.	Review sustainability data and identify priority projects for water

Within each theme, actions are ranked by the highest impact and lowest effort. Action numbers correspond to numbers presented in Figure 1C for cross-referencing.

**Table 5:** The list of actions in the *Plan* category of the *Core* tier, grouped by theme.

Theme	Number	Action
Team-identified objectives	1.	Outline a plan for achieving team-identified, objective goals for energy
	2.	Outline a plan for achieving team-identified, objective goals for procurement
	3.	Outline a plan for engagement with community on sustainability initiatives
	4.	Outline a plan for achieving team-identified, objective goals for waste
	5.	Outline a plan for achieving team-identified, objective goals for transportation
	6.	Outline a plan for achieving team-identified, objective goals for water

Within each theme, actions are ranked by the highest impact and lowest effort. Action numbers correspond to numbers presented in Figure 1D for cross-referencing.

**Table 6:** The list of actions in the *Energy* category of the *Clinic* tier, grouped by theme.

Theme	Number	Action
Energy efficient equipment	1.	Purchase equipment with favorable Energy Star or similar rating guide
	2.	Have a plan for investing in energy efficient equipment when replacement needed
	3.	Switch off appliances, lights, and appropriate equipment when not in use
	4.	Have an end-of-day checklist that includes appliances that can be switched off overnight
	5.	Automate shut down or standby when possible in equipment that can cope with a hard shut down
Energy efficient lighting	6.	Install LED lighting or certified energy-efficient lights
	7.	Install motion sensitive switching or timers
	8.	Use smart plugs or other devices to remotely time on–off periods
	9.	Post stickers or posters to encourage individuals to turn off switches
	10.	Adjust lighting levels to minimize energy use or use natural light when possible
Environmentally sustainable laundry protocols, following infection control guidelines	11.	Use a clothesline when possible and allowable
	12.	Combine or reduce number of loads
	13.	Utilize cool wash when possible and allowable
Enhanced HVAC efficiency	14.	Upgrade physical infrastructure (e.g., insulation, windows) that regulate heating/cooling
	15.	Install programmable thermostats for adjustment, e.g., lowering heating and reducing cooling when the office is empty
	16.	Perform regular maintenance and cleaning of air conditioning units to improve performance
	17.	Develop an energy-efficient heating and cooling protocol
	18.	Adjust thermostats seasonally
Renewable energy use	19.	Purchase off-site renewable energy sources from a green energy provider, when available
	20.	Generate renewable energy on-site
	21.	Install solar panels, if possible

Within each theme, actions are ranked by the highest impact and lowest effort. Action numbers correspond to numbers presented in Figure 2A for cross-referencing.

**Table 7:** The list of actions in the *Waste* category of the *Clinic* tier, grouped by theme.

Theme	Number	Action
Waste reduction	1.	Use paperless systems to communicate information to clients by phone or email
	2.	Consolidate or remove unneeded items from single-use and pre-packaged surgical trays
	3.	Restrict the use of gloves to essential usage
	4.	Repurpose used equipment when appropriate, uncontaminated and in compliance with waste regulations
	5.	Identify actions to reduce waste stream contamination
	6.	Update printing SOPs in hospital to default to double-sided printing
	7.	Remove color printing as an option
	8.	Use 100% recycled paper
	9.	Repurpose clean used syringes and lines for euthanasia, when possible and allowable
	10.	Use largest appropriate sharps bin to reduce plastic waste
	11.	Send clean, used fluid bags home as waterproof coverings for bandages/casts
	12.	Repurpose clean used fluid lines as ET ties
Reusable alternatives, following best practices and infection control standards	13.	Use reusable gowns
	14.	Use reusable textile surgical drapes
	15.	Use reusable sterilization wraps or metal tins
	16.	Reuse endotracheal tubes
	17.	Use OR-specific shoes instead of single-use shoe covers
	18.	Use reusable incontinence pads when applicable
	19.	Use reusable cloth surgical hats
	20.	Use reusable face masks
	21.	Restrict disposable mask use to sterile procedures
	22.	Utilize reusable sharps containers
	23.	Use reusable surgical hand towels
Recycling programs	24.	Educate the team on what can be recycled at the clinic
	25.	Implement a regionally informed recycling program, dependent on clinic’s waste stream and local recycling programs and regulations
	26.	Provide resources on e-waste recycling based on local regulations
	27.	Recycle used lead aprons appropriately when required
	28.	Provide resources on battery collection and recycling based on local regulations
	29.	Research specific unique recycling programs, e.g., toner cartridge recycling
	30.	Offer client facing recycling for pet food packaging
Anesthetic waste	31.	Update anesthetic protocols to prioritize use of lower carbon anesthetic techniques over high anesthetic gas use
	32.	Use lower flow, as appropriate to maintain a safe minimum flow for team, patient, and equipment
	33.	Eliminate mask or box inductions from protocols
	34.	Utilize regional anesthetic techniques effectively
	35.	Minimize long anesthetics
	36.	Leak check breathing systems and anesthetic machines daily
	37.	Perform regular maintenance of anesthetic equipment
	38.	Service anesthetic machines regularly
Pharmaceutical stewardship	39.	Use evidence-based antimicrobial stewardship guidance to mitigate resistance
	40.	Educate clients on proper disposal of medications and ensure clear guidelines for disposal are provided with dispensed medications
	41.	Prioritize oral medications over IV
	42.	Train staff on sustainable prescribing principles to reduce pharmaceutical waste
	43.	Use evidence-based protocols for environmental decision-making when prescribing medications
Other waste opportunities	44.	Offer resources on aquamation as an alternative to cremation for pet remains
	45.	Implement a program to compost or digest food waste
	46.	Use reusable items for kitchens/breakrooms
	47.	Use plant-based cat litter in clinic
	48.	Use bulk kitchen items rather than single use

Within each theme, actions are ranked by the highest impact and lowest effort. Action numbers correspond to numbers presented in Figure 2B for cross-referencing.

**Table 8:** The list of actions in the *Water* category of the *Clinic* tier, grouped by theme.

Theme	Number	Action
Indoor water conservation	1.	Provide resources on alcohol-based surgical skin preparation vs. traditional surgical scrub (e.g., Avogard, Sterilium)
	2.	Conduct annual bedding review to minimize microfiber plastic pollution
	3.	Utilize steam cleaners
	4.	Turn off the tap when using sinks
	5.	Implement and share resources on water conservation initiatives
	6.	Combine laundry loads
	7.	Provide best practice guidelines related to water conservation in clinic
	8.	Minimize dishwater loads in kitchen spaces
	9.	Post signage to promote water conservation in appropriate spaces
Water efficient equipment	10.	Install knee-on surgical sinks
	11.	Install motion-controlled faucets to non-surgical sinks
	12.	Add aerators to faucets
	13.	Regularly inspect water sources to reduce leakage, e.g., biannually
	14.	Install low-flow fixture devices
Outdoor water conservation	15.	Implement a rainwater or gray water collection system
	16.	Choose water-wise appropriate landscaping

Within each theme, actions are ranked by the highest impact and lowest effort. Action numbers correspond to numbers presented in Figure 2C for cross-referencing.

Our SMEs also provided extensive comments on the process, themes and individual actions. SMEs highlighted the challenge of balancing high-impact and resource-intensive interventions with smaller, team-driven behavioral changes to build momentum. While the current evidence base for many veterinary-specific sustainability actions is limited, there is growing focus and effort within this sphere. Across all domains, leadership buy-in and active team engagement emerged as the foundation of success, alongside considerations such as measurable targets and communication. These emerged as the primary drivers for clinic sustainability success from our SMEs. Long-term commitment and integrating sustainability into daily clinic operations were emphasized throughout as key to progress. Building on these overarching themes, the following sections summarize expert insights within the categories of the “Pathway to a More Sustainable Clinic” framework.

### 3.1 Core

The Core tier represents foundational, sustainability actions that establish the basis for more advanced interventions. Collectively, these actions highlight the importance of implementation, team empowerment, and leadership alignment as the backbone for progress in sustainability efforts. We had 29 actions in our final list in the Core tier (Figure 1) across the four categories. Convene had 17 actions and 4 themes (Table 2; Figure 1A), Assess had 10 actions and 2 themes (Table 3; Figure 1B), Reflect had 8 actions and 1 theme (Table 4; Figure 1C), and Plan had 6 actions and 1 theme (Table 5; Figure 1D). All actions within the Core categories are aligned with empowering the veterinary team to build clear goals and are thus presented without breakdown within categories.

SMEs emphasized that while creating a sustainability plan is foundational, meaningful impact depends on the comprehensive implementation of coordinated efforts rather than isolated actions. Success on sustainability initiatives depends on empowering a well-supported team with leadership buy-in, adequate resources, clear goals, and shared ownership across the entire clinic staff. While audits and data collection are essential starting points, their value was noted to be in driving follow-up. Engaging the team through gamification and friendly competition can be effective strategies to increase engagement and foster ownership. Ongoing communication can also help sustain momentum. Experts noted that sustainability must be embedded into clinical culture and operations to avoid becoming an afterthought or additional work in the already hectic world of veterinary clinical practice. Long-term commitment with consistent messaging, regular meetings, and integration into budgets and other areas across the clinic is key.

### 3.2 Clinic

The Clinic tier builds upon the core by focusing on clinic-level sustainability actions. Overwhelmingly, our literature review and SME process identified many more actions within Clinic (*n* = 124), relative to Core and Community. Clinic actions focus on physical and behavioral changes within specific categories of Energy (*n* = 21), Waste (*n* = 48), Water (*n* = 16), Procurement (*n* = 25), and Transportation (*n* = 14). Average effort and impact for actions in the Clinic category are presented in Figure 2. Each Clinic category is further discussed individually below.

#### 3.2.1 Energy

Energy actions aim to minimize energy usage and maximize energy from renewable sources. Our final list of actions in the Energy category was 21 actions and 5 themes (Table 6; Figure 2A). The goal of energy actions is to minimize energy usage and maximize energy from renewable sources. SMEs highlighted several key opportunities and limitations in reducing energy use. Experts noted that infrastructure improvements can yield significant energy savings but often come with high upfront costs and workload. For example, renewable energy use on-site was typically a medium impact action due to limitations in cost, availability, and capacity, while purchasing off-site renewable energy was considered easier to implement with maximal impact. Heating and cooling emerged as a significant clinical energy burden, with noted behavioral challenges in both implementing initial decreases in use, as well as needing to shift culture in order for behaviors to be sustained. Laundry practices, particularly reducing dryer use and using cold washes, were consistently cited as high-impact and feasible interventions. Behavioral actions, such as applying switch-off stickers to encourage turning off unused equipment, often have low effort with potential high impact. However, maintaining behavioral changes emerged as a major challenge. Within the comments, experts emphasized that while certain technical upgrades may have moderate effects with high initial cost, they collectively matter. Structural investment and continued demand for renewable and clean energy solutions can drive sustained change. Overall, expert comments on the energy category focused on a blend of technological upgrades, behavioral changes, and strategic purchasing to optimize impact.

#### 3.2.2 Waste

Waste actions focus on waste management protocols and opportunities to minimize clinical waste generation. The Waste action had the most actions (*n* = 48) among all Clinic categories, with 6 themes (Table 7; Figure 2B). For the Waste category, SMEs consistently emphasized the geographical specificity of waste management systems, the importance of anesthetic waste in veterinary medicine, and the need to prioritize carbon over physical waste. SMEs noted that while individual waste actions often have a low impact, their cumulative impact is meaningful. However, there was noted emphasis on the idea that waste, especially recycling, can serve as a distraction from the more meaningful high environmental impact areas. However, a strategic waste plan is both valuable for waste reduction as well as encouraging team engagement, as waste is one of the most visible aspects of sustainability and an entryway for interest. Waste audits may prompt further engagement in sustainable efforts in the clinic (15). Waste management was noted to be complicated by the diversity of clinic waste streams, as well as regulatory constraints associated with regional variation. Practical feasibility, infection control concerns, and staff buy-in must be balanced for success in this category already limited by current waste streams.

Experts noted that reusable alternatives to high-volume disposables, such as drapes and gowns, were promising, with effort and feasibility dependent on local availability. Behavioral strategies, from limiting glove use or reassessing sterile mask usage to something as simple as reducing printing, were cited as impactful with minimal effort. Anesthetic waste management in veterinary medicine emerged as the section with great potential impact, often with only moderate initial effort. Anesthetic protocols present opportunities for improving both sustainability and quality of care, with SMEs noting that lowest safe flow can have large impact. While several SMEs noted that greener anesthesia may improve care, it must be implemented safely and with evidence-based practices. Changes require staff training and careful implementation to avoid compromising patient outcomes. While resources for more sustainable veterinary anesthesia exist (16), there remains opportunities for learning, as many veterinary professionals are unaware about the impact of veterinary anesthesia (17). Other sections prompted discussion, such as aquamation where some experts noted the possible significant emission savings while others expressed concern about gaps in current evidence, as well as potential ecological impacts. Overall, experts emphasized the need for stronger evidence-based guidance for waste reduction practices.

#### 3.2.3 Water

Water focuses on minimizing water use with behavioral changes as well as equipment efficiency. Within the Water category, there were 16 actions and 3 themes (Table 8; Figure 2C). These included simple, but impactful, interventions. SMEs noted that laundry is a primary driver of water use in most clinics, reinforcing its relevance as an area for reassessment of sustainable protocols. Bedding choice was brought up as a concern due to differences in water use and production impact between cotton and recycled synthetic fabrics. Beyond total water use, there were additional comments about the impacts of sustainable protocols related to water, such as installing filters to minimize microplastic pollution from laundering. Experts again emphasized that reducing water use is dependent on geographic location. Water reduction actions may have mild impacts on carbon footprints, but there were many low effort, easy “wins” to significantly reduce water pollution and bring attention to sustainability.

#### 3.2.4 Procurement

Procurement focuses on making sustainable choices in clinic supplies and suppliers. The Procurement category had 25 actions and 2 themes (Table 9; Figure 2D). SMEs described procurement as an area where actions are often conceptually low effort with high impact, yet they are difficult in practice due to real-world constraints. Product availability, lack of supplier transparency, and managing purchasing in a clinic setting all emerged as challenges. Procurement decisions may also lie beyond the individual clinics’ control. A major source of discussion was Life Cycle Assessments (LCA), a tool for tackling Scope 3 emission. Scope 3 emissions are the indirect emissions generated across a clinic’s value chain, including the supply chain. While some experts mentioned the utility of LCAs, as well as emphasized the importance of asking suppliers to provide them, there remains minimal infrastructure for reliable LCA data and manufacturers can be unmotivated to reduce product-level impacts or provide LCAs. Still, actions for improving inventory management were highlighted for their waste-reducing potential. Even procurement choices were seen as impactful to help signal market demand and spark shifts in supply chains, even if individual impacts were moderate in the short term.

**Table 9:** The list of actions in the *Procurement* category of the *Clinic* tier, grouped by theme.

Theme	Number	Action
Clinic environmental procurement policy	1.	Prioritize reusable choices in purchasing decisions
	2.	Develop sustainable inventory management protocols
	3.	Purchase in bulk
	4.	Select low or zero carbon emission shipping options, if available
	5.	Consolidate orders and deliveries
	6.	Prioritize local and plant-based food choices for staff
	7.	Set stocking levels for products
	8.	Practice stock rotation
	9.	Prioritize purchase of products with third party certification
	10.	Mark open bottles with end-dates
Supply chain driven sustainable change	11.	Ask suppliers to use electric fleets
	12.	Choose reusable tote delivery programs
	13.	Ask shipping services for packaging take back programs
	14.	Ask suppliers to source 100% renewable electricity
	15.	Utilize shipping services that offer sustainable packaging
	16.	Participate in supply chain stakeholder sustainability discussions
	17.	Ask shipping services to use reduced or use recycled packaging
	18.	Ask suppliers for ice pack take back programs
	19.	Ask suppliers to provide life cycle assessment for all products
	20.	Ask suppliers to facilitate order consolidation
	21.	Ask manufacturers to use recycled content in products
	22.	Ask manufacturers to disclose or avoid use of chemicals of concern
	23.	Communicate your sustainable procurement policy with suppliers
	24.	Ask companies to share sustainability policies
	25.	Communicate strong preference for third party certifications

Within each theme, actions are ranked by the highest impact and lowest effort. Action numbers correspond to numbers presented in Figure 2D for cross-referencing.

#### 3.2.5 Transportation

Transportation focuses on using and promoting low emission transportation for the clinic, staff, and clients. The Transportation category had 14 actions and 2 themes (Table 10; Figure 2E). SMEs identified employee community and client transportation as significant contributors to veterinary-related emissions, accounting for an estimated 15% each of total footprints. Potential interventions are highly context dependent. Experts were skeptical about the feasibility of widespread carpooling or public transit use, especially in regions with limited infrastructure. Providing electric vehicle charging was seen as helpful to encourage transitions and showcase commitment to sustainability, but also limited by the number of charging stations to benefit staff charging and by the time required to benefit client charging. While shifting to electric vehicles and promoting public transit or cycling are important, infrastructural and behavioral barriers remain. There was noted discussion on the potential impact of telehealth. SMEs acknowledged limitations of current infrastructure and associated regulations, while noting facets of telehealth opportunities, for example reducing the number of recheck visits with phone calls or pictures. Overall, experts emphasized that transportation improvements are incredibly important, as the major contributor to a vet clinic’s carbon footprint, however deeply challenged by infrastructure limitations, as well as behavior changes, and may require supportive policies.

**Table 10:** The list of actions in the *Transportation* category of the *Clinic* tier, grouped by theme.

Theme	Number	Action
Clinic sustainable transportation	1.	Attend conferences virtually
	2.	Transition to low emission vehicles for owned and leased clinic vehicles
	3.	Install electric vehicle charging infrastructure for staff use
	4.	Subsidize monthly public transit passes for employees
	5.	Offer hybrid options for practice meetings
	6.	Create a structure for sign-ups to facilitate carpooling
	7.	Schedule shifts that facilitate carpooling
	8.	Provide lockers for bike commuter needs
	9.	Provide a safe place for people to store their bikes
	10.	Install showers for employee use
Client sustainable transportation	11.	Utilize telehealth and teleconsultation when appropriate
	12.	Reduce client mileage for prescription refills by using local pharmacies, if available and appliable
	13.	Reduce client mileage by consolidating appointments
	14.	Install electric vehicle charging infrastructure for client use

Within each theme, actions are ranked by the highest impact and lowest effort. Action numbers correspond to numbers presented in Figure 2E for cross-referencing.

### 3.3 Community

The Community tier focuses on engaging clients, staff, and the broader public in sustainability initiatives, leveraging education, partnerships, and advocacy to extend environmental impact beyond the clinic’s operations. Our final list of actions in the Community tier was 46 (Figure 3) across the five categories. Health had 13 actions and 3 themes (Table 11; Figure 3A), Stories had 8 actions and 2 themes (Table 12; Figure 3B), Connections had 6 actions and 3 themes (Table 13; Figure 3C), Policies had 9 actions and 2 themes (Table 14; Figure 3D), and Nature had 10 actions and 3 themes (Table 15; Figure 3E).

**Table 11:** The list of actions in the *Health* category of the *Community* tier, grouped by theme.

Theme	Number	Action
Access to care	1.	Provide preventive care packages
	2.	Contribute to low-cost services in your community
	3.	Have an internal fund to increase access to care
	4.	Share community resources to access veterinary care
Preventative healthcare	5.	Educate clients on the importance of preventive medicine in reducing the environmental impact of pet ownership (e.g., vaccination, heartworm prevention)
	6.	Provide resources on antimicrobial resistance for staff and client education (e.g., completing courses of antibiotics)
	7.	Identify and educate patients at high-risk from environmental stressors (e.g., brachycephalics and heat)
	8.	Connect clients to available resources on emergency planning/disaster preparedness for pets
	9.	Share resources on environmental hazards impacting patient and client health (e.g., air quality, heat)
Sustainable diagnostics and therapeutics	10.	Reserve diagnostic tests for those that are necessary or will alter the treatment plan
	11.	Use evidence-based approaches matched to the patient for drug prescriptions to avoid overprescribing
	12.	Use lower impact treatment options
	13.	Consider medication route when selecting treatment options (e.g., oral over IV)

Within each theme, actions are ranked by the highest impact and lowest effort. Action numbers correspond to numbers presented in Figure 3A for cross-referencing.

**Table 12:** The list of actions in the *Stories* category of the *Community* tier, grouped by theme.

Theme	Number	Action
Sustainability achievements	1.	Communicate sustainability initiatives and achievements internally with staff and leadership
	2.	Communicate sustainability initiatives and achievements to clients
	3.	Communicate sustainability initiatives and achievements at a community level
Sustainability education and promotion	4.	Educate staff on the connection between sustainability initiatives and health
	5.	Lead rounds on sustainability practices
	6.	Educate clients on the animal health impact of environmental hazards
	7.	Promote sustainability activities in the community
	8.	Talk at a local school about veterinary medicine and sustainability

Within each theme, actions are ranked by the highest impact and lowest effort. Action numbers correspond to numbers presented in Figure 3B for cross-referencing.

**Table 13:** The list of actions in the *Connections* category of the *Community* tier, grouped by theme.

Theme	Number	Action
Sustainability networks and recognition programs	1.	Participate in clinical sustainability networks
	2.	Participate in sustainability recognition programs outside of clinical medicine
Health professional engagement	3.	Present on sustainable topics at conferences
	4.	Write a sustainability focused veterinary communication
	5.	Request sustainability topics at conferences
Community engagement	6.	Engage with sustainability at the local and regional level of your community

Within each theme, actions are ranked by the highest impact and lowest effort. Action numbers correspond to numbers presented in Figure 3C for cross-referencing.

**Table 14:** The list of actions in the *Policies* category of the *Community* tier, grouped by theme.

Theme	Number	Action
Local level advocacy	1.	Encourage national veterinary medical associations to launch sustainability initiatives
	2.	Support local action on sustainability topics
	3.	Contact local or regional representatives
	4.	Participate in local veterinary medical associations
	5.	Attend city council meetings
Government involvement	6.	Support staff voting in local, regional and national elections (e.g., provide time for staff to vote)
	7.	Advocate for animal health internationally (e.g., support international animal health NGOs)
	8.	Advocate for animal health in regional or national government
	9.	Advocate for animal health in local government

Within each theme, actions are ranked by the highest impact and lowest effort. Action numbers correspond to numbers presented in Figure 3D for cross-referencing.

**Table 15:** The list of actions in the *Nature* category of the *Community* tier, grouped by theme.

Theme	Number	Action
On-site biodiversity	1.	Eliminate use of ecotoxic treatments
	2.	Choose regionally appropriate biodiverse landscaping
	3.	Plant a clinic pollinator garden
Community biodiversity	4.	Encourage team participation in natural area clean-ups
	5.	Use social media to engage with local biodiversity efforts
	6.	Promote local community activities
	7.	Hang flyers or posters in clinics for local events
Global biodiversity	8.	Promote global biodiversity initiatives to staff, clients, community
	9.	Provide resources to support global biodiversity initiatives
	10.	Donate time or money to support biodiversity initiatives

Within each theme, actions are ranked by the highest impact and lowest effort. Action numbers correspond to numbers presented in Figure 3E for cross-referencing.

SMEs highlighted actions within the broad community category with the lens of veterinarians as a profession uniquely positioned as climate communicators with a shared concern for animal health. However, turning this potential into real impact was seen as difficult when requiring training and leadership support for climate conversations. Efforts to increase community access to care or offer low-cost services were noted to face significant constraints, especially in hospital models where economic pressures may conflict with sustainability goals. Preventive care was identified as the area with the maximal impact; however, experts again noted the lack of low-impact evidence-based diagnostic and treatment recommendations. Experts emphasized that engagement can be more impactful than specific environmental actions themselves. Finally, while national and global sustainability efforts were seen as valuable, most SMEs recommended focusing locally to maximize impact within the community. Veterinarians hold a trusted voice in their communities and have a unique opportunity to start climate action conversations with clients. Community highlights the unique role of veterinarians as trusted messengers and the complexity of embedding sustainability within practice culture. Veterinarians’ role as educators and advocates for environmental and public health must be within the clinic and beyond its walls. Community engagement is a promising, yet emerging, avenue for advancing sustainability in veterinary clinics.

## 4 Discussion

The results of this project provide veterinary clinics and teams with a practical starting point for engaging in environmental sustainability. By offering a range of nearly 200 actions, from highly visible and impactful to small zero-cost changes, clinics can choose where and how they want to start their sustainability journey. The actions provide sustainability teams ideas for paths forward for their clinic, or ways to connect people to sustainable opportunities. Even actions with moderate impact can be entry points to spark interest and get people in the sustainability door. Quick wins can help create buy-in and momentum for longer-term change. Our goal was to provide sustainable resources to those who want them and allow clinics to get involved with sustainability immediately. Clinics can choose the initiatives that fit their goals and their resources, increasing their impact over time. Every veterinary clinic can start somewhere, and having accessible, evidence-informed resources on hand empowers veterinary professionals to take immediate, meaningful steps toward embedding sustainability into practice.

Beyond informing veterinary professionals in practice, the findings from this project can inform the advancement of educational programming on environmental sustainability in veterinary schools and provides the opportunity for teaching institutes to model best practices. In 2023, the American Association of Veterinary Medical Colleges released a position statement on climate change, encouraging member institutions to integrate climate change and related topics into their curricula (18), and an AAVMC working group has since been formed to support educators in rapidly developing and embedding relevant content. The themes and actions identified in this study provide a practical starting point for creating educational resources that both highlight the breadth of possible interventions and emphasize those with the greatest impact. These materials could be incorporated into veterinary curricula, continuing education, and professional development programs focused on practice management. As sustainability is a relatively new focus in the profession, more senior veterinary professionals are less likely to have been introduced to the topic during their training (3), making continuing education particularly important. Such programming should also be accessible to veterinary paraprofessionals, a group with demonstrated interest in the topic and a track record of championing and implementing these changes in clinics (6). Integrating these best practices into teaching hospital operations in both the U.S. and Canada would immerse trainees in sustainable workflows from the outset, fostering not only the adoption of environmentally responsible behaviors but also positioning veterinary professionals as credible advocates for sustainability within their communities (5, 19). These resources may also support building a foundation for veterinarians to play a more active role in science communication. All veterinary professionals deserve to have access to continued learning on sustainability and communication on veterinary sustainability throughout their careers.

This project could also shape change in the broader veterinary industry. These actions provide a clear roadmap for environmentally sustainable clinic operations and could serve as a foundation for developing a third-party certification for veterinary clinics. Third-party certifications, awarded by independent organizations, verify that products, services, or facilities meet established standards. In human healthcare, non-profit organizations exist to provide guidance, progress assessments, and recognition to providers that have decarbonized their operations (9, 10). Although veterinary-specific certifications or awards exist in other countries (20, 21), to our knowledge, no comparable certification exists for veterinary practices in the U.S. or Canada. This gap is notable given that more than 65% of surveyed U.S. veterinary clients indicated they would value a certification identifying clinics that have reduced their carbon footprint (8). A veterinary-specific certification would also promote further research for vet clinics to minimize carbon emissions (22). The list of actions developed in this study could guide a certifying body in establishing such a program. Like existing models, certification could be tiered (e.g., bronze, silver, gold, platinum), allowing clinics to progress stepwise. Lower tiers might reward “easy wins” requiring minimal resources, while higher tiers would recognize more resource-intensive, higher-impact measures. Such a certification would be mutually beneficial to both clinics and clients. Public recognition can appeal to environmentally conscious clients, thereby attracting additional business. Additionally, the certification process can serve as a structured framework for clinics to measure progress and identify goals for continued improvement.

While this study establishes a foundation of best practices for environmental sustainability in veterinary medicine, significant opportunities remain for further research. All our SMEs highlighted the lack of objective data on the actual environmental, economic, and operational impacts of these actions when implemented in veterinary settings. In some cases, evidence can be extrapolated from human healthcare (e.g., anesthesia) or related sectors; however, there is a need for veterinary-specific information in other cases (e.g., companion animal aquamation). More broadly, robust, veterinary-specific data would strengthen the case for adoption, allow prioritization of high-impact interventions, and inform cost–benefit analyses relevant to diverse practice types. Equally important is research on behavior change in the veterinary profession, identifying the social, cultural, and organizational factors that facilitate or hinder the uptake of sustainable practices. This includes exploring effective communication strategies, incentives, and leadership models that can shift norms and embed sustainability into routine clinical operations. Such work would not only close key knowledge gaps but also provide the evidence-based needed to drive measurable, lasting change in the profession.

Continued research will be important to address the limitations of this study. Our literature review was restricted to freely accessible, English-language resources, which may have excluded relevant actions described in subscription-based publications or in other languages. Likewise, all members of our SME panel were native English speakers, introducing the possibility of similar language bias in the expert review process. Our SME panel represented primarily small animal practices, limiting the scope of practice type represented. While we believe our SME panel was highly qualified, the emerging nature of veterinary sustainability means that the current pool of experts is small, potentially limiting the diversity of perspectives represented. As the evidence base expands and the field matures, it will be important to revisit and refine the list of themes and actions presented here, using this initial framework as a foundation for incorporating new information, experiences, and stakeholder input.

## 5 Conclusion

Through a multi-phase, expert-informed process, we collaboratively developed practical actions for environmental sustainability best practice in veterinary hospitals and clinics in the US and Canada. Environmental sustainability in veterinary medicine is influenced by a range of factors, including geographic location, resource availability, and clinic-specific goals. Despite this variability, we identified hundreds of actions that clinical teams can adopt to reduce the environmental impacts associated with veterinary care delivery. These actions reflect the nuanced challenges and opportunities identified by SMEs and ground each recommendation in real-world clinic contexts. Importantly, these actions are not a one-size-fits-all prescription, but rather a flexible framework that clinics can adapt based on their size, location, resources, and sustainability goals. Additionally, the effort and impact matrix supports clinics beginning their sustainability journey by providing low-cost, high-visibility actions, while facilitating progression toward more resource intensive actions that have higher impact measures. By integrating clinical practice with environmental stewardship, this list supports veterinary teams in delivering high-quality care while contributing meaningfully to planetary health. We hope it serves as both a roadmap and a catalyst for action to help clinics start, sustain, and scale their sustainability journeys.

## Data Availability

The original contributions presented in the study are included in the article/Supplementary material, further inquiries can be directed to the corresponding author.
